# Can adjuvant chemotherapy improve the prognosis of adult ovarian granulosa cell tumors? A narrative review

**DOI:** 10.1097/MD.0000000000029062

**Published:** 2022-03-18

**Authors:** Yuan Zhuang, Shushan Zhang, Yao Liu, Hua Yang

**Affiliations:** ^a^ *Department of Gynecology, The Fifth Affiliated Hospital of Sun Yat-sen University, Zhuhai, China,* ^b^ *Sun Yat-sen University, Zhuhai, China.*

**Keywords:** adjuvant chemotherapy, adult granulosa cell tumor, advanced stage disease, prognosis, recurrent disease

## Abstract

Adult granulosa cell tumors (aGCTs) are rare ovarian neoplasms with a relatively favorable prognosis. They follow an indolent course, characterized by a prolonged natural history and a tendency to late recurrences, Around a quarter of patients develop recurrence and More than 70% of women with recurrence die from their disease, The percentage of patients received chemotherapy increases over time, whether adjuvant chemotherapy improve the prognosis of aGCTs is equivocal? The purpose of this review is to summarize the previously published evidence to evaluate whether adjuvant chemotherapy improve the prognosis of aGCTs to provide guidance for clinical practice. EMBASE, PubMed, Web of Science, WanFang Data and Chinese National Knowledge Infrastructure are searched up to December 2020, used the search strategy of ovar* and granulosa cell* and (tumor* or tumour* or malignan* or cancer* or carcinom* or neoplasm*) and chemotherapy. The screening process was conducted strictly based on inclusion and exclusion criteria. Clinical studies based on human including randomized controlled trial, quasi-randomised controlled trials, nonrandomised trials cohort study and case control study were included without restriction of time. The percentage of patients received chemotherapy increases over time, but the benefit of adjuvant chemotherapy is lack of high-grade evidence of prospective study, based on the current retrospective studies, we still do not have the evidence to confirm the survival benefit of adjuvant chemotherapy in early stage, advanced stage or recurrent aGCT with no residual tumor, but for inoperable disseminated disease or disease with suboptimal cytoreduction, adjuvant chemotherapy maybe an Optable options. Multinational prospective randomised controlled trials are urgently needed to validate the role of adjuvant chemotherapy. Further research on molecular mechanisms and developing novel targeted medicines may improve the survival of aGCTs.

## 1. Introduction

Granulosa cell tumors (GCTs) are rare ovarian neoplasms. They were described for the first time in 1855 by Rokitansky.^[1]^ They usually occur in the peri- and postmenopausal period with peak prevalence in patients aged 50 to 55 years. The incidence is around 0.47 to 1.6 per 100,000, accounts for 2% to 5% of all ovarian cancers,^[2,3]^ is the most common sex cord-stromal tumor and can be divided into 2 subtypes according to the differences of the age of patients, clinical and histopathologic features. About 95% of GCTs belong to the adult granulosa cell tumors (aGCTs), and others are juvenile GCTs.^[4]^ The main risk factors of GCTs include nulliparity, fatness, oral contraceptives, and family cancer history. The symptoms are various: abdominal pain (30%-50%), abdominal distension related to mass effect, and hormonal events (41%) such as irregular menstruation, intermenstrual bleeding, postmenopausal bleeding or amenorrhea.^[5]^ These tumors are malignancies with a relatively favorable prognosis^[6]^ characterized by a prolonged natural history and a tendency to late recurrences.

aGCTs are clinical and molecular unique subtype of ovarian cancer, characterized by a pathognomonic somatic missense point mutation 402C->G (C134W) in the transcription factor fork head box protein L2 (FOXL2).^[7]^ The FOXL2 402C->G mutation leads to increased proliferation and survival of granulosa cells, and promotes hormonal changes. Histological diagnosis of adult-type granulosa cell tumor is challenging, therefore testing for the FOXL2 mutation is crucial for differential diagnosis,^[8-10]^ anti-Mullerian Hormone^[11]^ and inhibin B^[12]^ are currently the most accurate circulating biomarkers. The majority of aGCTs are diagnosed at an early stage, have an excellent prognosis. However, around a quarter of patients develop recurrent tumors and require further treatment, one-third of recurrences occur more than 5 years after initial treatment. Reported 5-year overall survival (OS) for patients with stage I disease ranges from 75% to 95%.^[6,13-15]^ However, the 5-year OS rate is reduced to between 55% and 75% for patients with stage II tumour and dropped to 22% to 50% for those with stage III/IV tumour. The reported overall 10-year survival rate ranges from 85% to 95%, and disease mortality rate is approximately 20%. More than 70% of recurrent patients die from the disease.^[16,17]^ Surgery is the cornerstone for the treatment of both primary and relapsed tumor,^[18-20]^ although National Comprehensive Cancer Network (NCCN) guidelines^[21]^ recommend adjuvant chemotherapy for patients with advanced stage disease, or stage I disease with high risk factors. The rarity, indolent behavior and tendency to late relapse make it difficult to conduct well-designed randomized studies to define the value of this strategy. The use of adjuvant chemotherapy has sometimes been associated with prolonged disease-free survival (DFS) and possibly OS.^[22,23]^ Although the overall rate of response is high,^[24,25]^ whether adjuvant chemotherapy improves the prognosis of aGCTs is equivocal. The purpose of this review is to summarize the previously published evidence to evaluate whether adjuvant chemotherapy improve the prognosis of aGCTs to provide guidance for clinical practice.

## 2. Methods

Papers in all languages were sought and translations carried out when necessary. The electronic databases EMBASE, PubMed, Web of Science, WanFang Data, and Chinese National Knowledge Infrastructure were searched up to December 2020, and the “related articles” feature was used to carry out a further search for newly published articles. A Google search was conducted to look for Internet based resources and openaccess publications search strategy: ovar* and granulosa cell* and (tumor* or tumour* or malignan* or cancer* or carcinom* or neoplasm*) and chemotherapy. For completeness, we searched for relevant randomised controlled trials (RCTs) and quasi-RCTs, nonrandomised trials, prospective and retrospective cohort studies, case-control studies. Case report and case series were excluded. The flow chart is shown in the Figure 1.

**Figure:**
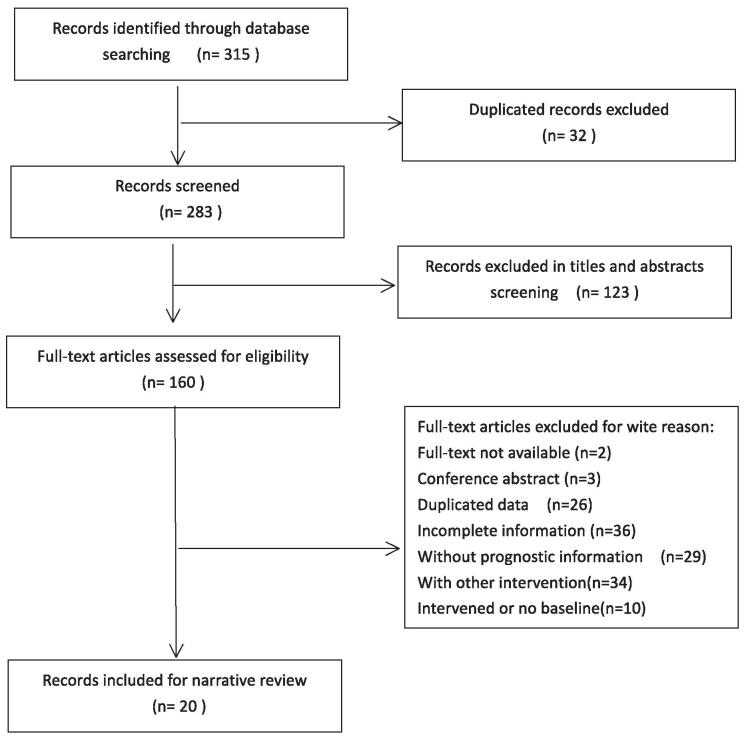
**Figure 1.** Flow diagram for the assessment of studies identified in the narrative review.

## 3. Results

All titles and abstracts retrieved by electronic searching were downloaded to the reference management database Endnote, duplicates were removed and remaining references were examined by 2 review authors (ZY, ZSS). Those studies that clearly did not meet the inclusion criteria were excluded, and copies of the full text of potentially relevant references were obtained. The eligibility of retrieved papers was assessed independently by 2 review authors (ZY, ZSS). The 19 included retrospective cohort studies and 1 systematic reviews provided prognostic data of chemotherapy on 5148 women with aGCTs; 2 review authors (ZSS, LY) independently abstracted data from each included study using a specially designed data collection form, with differences resolved by discussion or by a third review author when necessary (YH).

## 4. Discussion

### 4.1. Prognostic factors in aGCTs

The efficacy of prognostic factors in aGCTs had been taken from a number of retrospective studies. Various factors showed to have prognostic significance included age, tumor size, rupture of tumor, postoperative residual tumor, nonstaging surgery, mitotic activity, nuclear atypia, aneuploidy, p53 over-expression, high Ki-67, and stage of the disease.^[14,26-30]^ Only stage of the disease had been consistently shown in various studies to affect survival. In recently study by Park et al,^[31]^ the 5 year DFS and OS rates in early stage (stage I and II) disease was 89% and 99%, respectively while in advanced stage (stage III and IV) disease it was 72% and 80%, respectively. The 10 year DFS and OS rates in early stage (stage I and II) disease was 89% and 90%, respectively while in advanced stage (stage III and IV) disease it was 57% and 67%, respectively.

The somatic missense mutation in the FOXL2 gene (c.402 C>G;p.C134W)l,^[8]^ which was found in approximately 97% of aGCTs argues strongly that this mutation has an etiologic role in these tumors. Because virtually all aGCTs contain the FOXL2 mutation, it would seem logical that recurrence and/or aggressive behavior would reflect other subsequent somatic genetic changes in the tumor. Park et al^[31]^ performed array comparative genomic hybridization experiments and FOXL2 genotyping by allelic discrimination on 40 tumor samples suggested that the acquisition of FOXL2 homozygous genotype was likely to be involved in tumor recurrence, and might be a marker of early recurrence. Various signaling pathways had been explored in the development of aGCTs, included transforming growth factor beta,^[3]^ phosphatidylinositol-3-kinase serine/threonine kinase,^[32]^ GATA4,^[33]^ and vascular endothelial growth factor.^[34-36]^ These factors had been shown to play important roles in granulosa cell proliferation, apoptosis or angiogenesis.

Alexiadis et al^[37]^ used whole exome and targeted telomerase reverse transcriptase (TERT) promoter sequencing found although aGCTs were defined by the presence of a common FOXL2 gene mutation, recurrence and/or aggressive behavior cannot be attributed to subsequent mutation of specific gene or pathways; however, there was a high frequency of the TERT-124C>T promoter mutation, which was associated with more aggressive disease. Da Cruz Paula et al^[38]^ tested the genomic profiling of primary and recurrent aGCTs, suggested that aGCTs were genetically heterogeneous tumors and that TERT promoter mutations and/or genetic alterations affected other cell cyclerelated genes might be associated with disease progression and recurrences.

Na et al^[39]^ analyzed the immunohistochemical p16 expression in the peri-tumoral stroma of primary and recurrent aGCTs, found primary aGCTs had significantly higher stromal p16 expression levels than nonpathological ovaries and benign sex cord-stromal tumors, recurrent aGCTs showed significantly elevated levels of stromal p16 expression compared to primary tumors, suggested stromal p16 expression might be involved in the development and progression of aGCTs.

Hillman et al^[40]^ used whole-exome and cancer gene panel sequencing of 79 aGCTs, found truncating mutation of the histone-lysine N-methyltransferase 2D (KMT2D; also known as MLL2) was a recurrent somatic event in aGCTs. Mono-allelic KMT2D-truncating mutations were more frequent in recurrent (10/44, 23%) compared with primary (1/35, 3%) aGCTs. immunohistochemical detected additional non-KMT2D-mutated aGCTs with loss of nuclear KMT2D expression, suggested that nongenetic KMT2D inactivation might occur in this tumor type. These findings identified KMT2D inactivation as a novel driver event in aGCTs and suggested mutation of this gene might increase the risk of disease recurrence.

Guleria et al^[41]^ outlined histomorphological spectrum of aGCTs and emphasized its frequent occurrence in lower stages with late recurrences, found histomorphological features were not prognostically important. However, prognostic value of Ki-67 couldn’t be excluded.

Färkkilä et al^[33]^ assessed clinicopathological prognostic factors and the prognostic roles of the human epidermal growth factor receptors (HER 2-4) and the transcription factor GATA4 in aGCTs, found high-level expression of HER2, and GATA4, and high nuclear atypia were prognostic of shorter DFS. Anttonen et al^[42]^ tested the role of factors regulating the normal granulosa cell function in 80 cases, found the more aggressive aGCTs retained a higher GATA-4 expression, concluded HER2 and GATA4 were new molecular prognostic markers of aGCTs recurrence, could be utilized to optimize the management and follow-up of patients with earlystage aGCTs

Roze et al^[43]^ investigated potential target-able genomic alterations by whole genome sequencing on 46 tumor samples and matched normal DNA, found higher mutational burden in recurrent tumors, and identified tumor protein p53 (TP53) mutations in 3 patients (3/33; 9.1%) with higher mitotic activity. They proposed that tumors with TP53 mutations defined a highgrade subgroup of aGCTs and might respond better to chemotherapy than patients without this variant.

Da Cruz Paula et al^[38]^ used targeted sequencing of over 400 cancer-related genes on a cohort of 38 aGCT patients. They identified mutations present in recurrent aGCTs that were not present in primary aGCT, including TERT promoter mutations (C228T and C250T), MED12, and TP53, as well as CDKN2A/B homozygous deletions, suggested that genetic alterations in cell cycle-related genes might be associated with recurrence.

Sakr et al^[44]^ evaluated novel predictors of recurrence in GCTs from a single institution cohort of 125 case between 1975 and 2014, found for early-stage (stage I) aGCTs, high expression of SMAD3 was a independent predictors of recurrence.

Jurí et al^[45]^ studied proliferative indices and microvessel density of aGCTs confirmed the importance of proliferative factors, tumor size, and histologic patterns as possible prognostic indicators for estimating the biologic behavior. Unfortunately, angiogenesis did not seem to be a useful determinant parameter of a possible aggressive behavior.

The development of aGCTs involved a variety of cell signaling pathways, these signal pathways made up a complex network and contributed to the formation and development of aGCTs. Future research should further focus on exploring the molecular mechanisms of aGCTs and developing novel targeted medicines to prevent recurrent of aGCTs.

### 4.2. The survey of adjuvant chemotherapy in aGCTs

Although there is a lack of high-grade evidence of prospective study to approve the benefit of adjuvant chemotherapy, the percentage of receiving chemotherapy increased over time; Nasioudis et al^[46]^ retrospectively analysed stage IC aGCTs from The National Cancer Database between 2004 and 2015 and found the percentage increased from 22.7% to 31.5% to 36.2% and 39.2% over each respective 3-year interval from 2007 to 2015. Several regimens of chemotherapy were effective in clinic study.

Pecorelli et al^[47]^ investigated the clinical activity and toxicity of a modified PVB regimen (cisplatin, vinblastine, and bleomycin) in patients with advanced or recurrent, pure granulosa cell tumours (GCTs) or mixed granulosa-theca cell tumours (GTCTs) (an EORTC Gynaecological Cancer Cooperative Group study), 38 eligible patients were entered in this trial, In the group of 25 patients those had received prior surgery only, 7 and 6 patients had complete and partial responses (PRs), respectively (response rate: 52%). In the group of 13 patients who had previously received postoperative radio- or chemotherapy, 5 complete and 5 PRs were observed (response rate: 77%). with a median duration of response of 20 months for both groups, confirmed the therapeutic activity of the PVB regimen in advanced/recurrent aGCTs. Colombo et al^[48]^ treated 11 women with recurrent and/or metastatic aGCTs with PVB regimen, 6 pathological completely responded, 3 partially responded (response rate: 81.8%). Zambetti et al^[49]^ treated 7 consecutive patients with advanced/recurrent aGCTs with PVB; 3 complete and 1 PR were observed, for an over-all response rate of 66%, and confirmed the therapeutic activity of the PVB regimen.

To reduce the toxicity with PVB, vinblastin was replaced with etoposide. Homesley et al^[50]^ assessed efficacy and toxicity of the combination of bleomycin, etoposide, and cisplatin (BEP) in Phase II trial as first-line therapy for ovarian stromal malignancies (a Gynecologic Oncology Group study). Seventy-five women were entered; 37% (14/38) of the patients undergoing secondlook laparotomy had negative findings. The 6 complete responders were of long median duration (24.4 months). Pautier et al^[51]^ investigated the activity and toxicity of BEP regimen in aGCTs in 20 consecutive patients with initial metastatic (5 patients) or recurrent (15 patients). The overall response rate was 90% (9 clinical complete response [CR], 9 clinical PR) with a median duration of 24 months (range: 4-77). At 4 years, OS and event-free survival were 58% and 30%, respectively. BEP regimen appeared to be an active regimen for aGCTs in firstline chemotherapy.

Chiara et al^[52]^ treated 9 chemotherapy-naive women with recurrent (2 patients) or high risk factors aGCT with cisplatin, cyclophosphamide with or without doxorubicin or cisplatin, etoposide, and bleamycin (PVP-16B), clinical CR was achieved in the 2 patients with recurrent disease. Five patients underwent second look surgery which documented: CR in 3 patients, PR in 1 patient, and progressive disease in 1 case. Median survival was 85 months (range 14-103). Pectasides et al^[53]^ valued the efficacy of PAC in 10 cases of advanced or recurrent aGCTs, 5 CRs and 1 PR were obtained for a total response rate of 60%. PAC regimens might be of benefit in the treatment of recurrent or high risk aGCTs.

Unfortunately etoposide was myelotoxic with a small risk of secondary acute myeloid leukemia. Brown et al^[54]^ compared the efficacy and side effects of taxanes, with or without platinum to BEP in treating sex cord-stromal ovarian tumors. In newly diagnosed patients, there was no significant difference in response rate, progression-free survival (PFS) or OS. Among patients treated for recurrent measurable disease, the response rate was higher for BEP-treated (71%) than for taxane-treated patients (37%), but this was not statistically significant. Taxanes demonstrated activity against sex cord-stromal tumors of the ovary and might be less toxic. Gynecologic Oncology Group (GOG) 187, a phase II study of paclitaxel for ovarian stromal tumors as first-or second-line therapy, GOG-264 compared BEP to carboplatin and paclitaxel were now complete and in followup; While this might alleviate the morbidity of adjuvant treatment, it could not answer the question of whether there was benefit for adjuvant chemotherapeutic treatment in aGCTs, unless 1 treatment arm demonstrated significant improvement in survival over another.

Many prior studies suggested that there were clinical activity associated with chemotherapy in aGCTs, it might not be enough to produce long-term responses. One study^[55]^ of 7 patients treated with BEP for metastatic sex cord stromal tumor reported responses in 6 patients (83%), but only 1 patient (14%) had a durable remission. Another study^[56]^ of patients with incompletely resected stages II to IV disease found that while 11 patients (69%) with advanced-stage primary tumors and 21 (51%) with recurrent disease remained progression-free at the end of the study. Many of these had stable disease or nonmeasurable response rather than definitive tumor shrinkage. In general, some studies suggested that women with advancedstage or recurrent disease achieve clinical benefited from postoperative chemotherapy, but others failed to show that the use of chemotherapy was associated with better PFS or OS. As a result, practice was highly variable. Some centers recommended postoperative chemotherapy for all women with newly diagnosed stage IC to IV disease, most often with BEP or other platinumbased chemotherapy such as paclitaxel and carboplatin; others recommended chemotherapy only for women with residual disease after surgery. Still others did not recommend initial chemotherapy for any stage of disease but only at the time of recurrence.

### 4.3. Whether adjuvant chemotherapy improves the prognosis of early stage aGCTs?

Patients with early stage disease (stage I and II) have good prognosis, with 5 year DFS and OS of 89% and 99%, respectively^[31]^ and these patients usually don’t require any postoperative treatment. Patients with stage IC accompanied with poor prognostic factors and stage II have a higher chance of relapse, may benefit from postoperative treatment but the role of chemotherapy is still debatable. Current NCCN guidelines^[21]^ recommends adjuvant chemotherapy for stage I disease with high risk factors, but clear definition of what constitutes a high risk factor remains controversial. The main outcome of studies related to adjuvant chemotherapy in early stage aGCTs was summarized in Table 1.

**Table 1:** The main outcome of studies related to adjuvant chemotherapy in early stage aGCTs.

**Reference**	**Year**	**Type of research**	**Sample**	**Outcome**
Babarovic’ et al^[57]^	2018	Retrospective	36	Not reported
Oseledchyk et al^[58]^	2018	Retrospective	739	Not associated
Mehta et al^[59]^	2005	Retrospective	40	Not associated
Meisel et al^[60]^	2015	Retrospective	118	Not associated
Mangili et al^[61]^	2016	Retrospective	40	Not associated
Chan et al^[62]^	2005	Retrospective	83	Not associated
Lee et al^[14]^	2011	Retrospective	102	Not associated
Nasioudis et al^[46]^	2019	Retrospective	427	Not associated
Wang et al^[63]^	2018	Retrospective	60	Not associated

Babarovic’ et al^[57]^ analyzed clinical and pathohistological parameters and their impact on recurrence, overall and DFS in Federation International of Gynecology and Obstetrics (FIGO) stage I aGCTs patients, found FIGO substage IC was predictive of recurrence and DFS in patients with early-stage aGCTs. LVSI, presence of necrosis and hemorrhage, diffuse growth pattern, and nuclear atypia seemed to be associated with overall and DFS, because the low rate of adjuvant chemotherapy, the researcher couldn’t draw any meaningful conclusions regarding the role of adjuvant chemotherapy.

Oseledchyk et al^[58]^ assessed the outcomes of aGCTs from the surveillance, epidemiology, and end results database between 2000 and 2013. The largest retrospective study, thus far that included a total of 739 patients with 570 stage I and 139 substage IC patients, also failed to detect an association between adjuvant chemotherapy and improved DFS in any of the subgroups or in the entire study cohort.

Mehta et al^[59]^ retrospectively studied 40 cases of aGCTs, majority of patients presented with FIGO stage I. 62.1% of aGCTs were given postoperative chemotherapy, but postoperative chemotherapy, mitosis or histological patterns were of little significance.

Meisel et al^[60]^ undertook a systematic review of the large number of patients treated for this disease at Memorial Sloan Kettering Cancer Center from January 1996 through June 2013 with the goal of further defining the role for chemotherapy, during stage I. Those who did not receive adjuvant therapy had a significantly longer median PFS and a significantly higher 3-year PFS rate than those who received adjuvant therapy (*P*<.001). Adjuvant chemotherapy did not improve the recurrence-free interval, given the significant short- and longterm toxicities associated with the chemotherapy, observation following complete surgical resection might be a reasonable option.

Mangili et al^[61]^ assessed the efficacy of first line postoperative chemotherapy in patients with stage IC treated at the Italian Centers involved in the multicenter Italian trials in ovarian cancer group. A total of 40 patients with primary GCTs of the ovary at FIGO stage IC were identified, but adjuvant chemotherapy did not retain significant predictive value for recurrence.

Chan et al^[62]^ analysed prognostic factors responsible for survival in sex cord stromal tumors of the ovary, enrolled 83 women with Sex Cord Stromal Tumor of the ovary, including 73 with granulosa and 10 with Sertoli-Leydig cell tumors. Fifty-one were stage I patients who received adjuvant chemotherapy and did not impact survival.

Lee et al^[14]^ conducted a multi-center retrospective study and enrolled 102 patients of aGCTs, 86 patients at stage I. In multivariate analysis, stage was the only factor associated with recurrence; adjuvant chemotherapy was not statistically significant.

Nasioudis et al^[46]^ evaluated the role of adjuvant chemotherapy in the management of stage IC aGCTs of National Cancer Data Base between 2004 and 2015. A total of 427 patients were included in the survival analysis. The median follow-up of the chemotherapy (n = 145) and observation (n = 282) groups were 57.3 and 61.5 months, respectively. A total of 11 (7.6%) and 29 (10.3%) deaths were observed in the chemotherapy and observation groups, respectively. Following the generation of Kaplan-Meir curves, 5-year OS rates were 93.7% and 91.6%, respectively and there was no difference in OS observed between the 2 groups (*P* = .52); after adjusting for patient age (<50 vs ≥50 years), tumor size (<10 vs ≥10cm vs unknown), and the performance of lymph node dissection, the administration of adjuvant chemotherapy was not associated with a survival benefit (hazard ratio: 1.07, 95% confidence interval: 0.52-2.21).

Wang et al^[63]^ performed a retrospective study of patients with stage IC aGCTs diagnosed at Peking Union Medical College Hospital from January 1985 to September 2015. Sixty stage IC aGCTs patients were identified, including 28 in the no adjuvant chemotherapy group (ACG) and 32 in the ACG. The 5-year DFS rates in the no ACG and ACG groups were 76.3% and 87.5%, respectively (*P* = .197). Adjuvant chemotherapy was thus not associated with improved DFS.

Due to the rarity of aGCTs, these data have been collected from retrospective studies and it’s difficult to conduct a randomized controlled trial to assess the efficacy of postoperative treatment in high-risk patients. Thus we still don’t have the evidence showing postoperative treatment in the adjuvant setting can confer a survival benefit in high risk patients. The potential serious toxicity of chemotherapy with a lack of obvious benefit suggests comprehensive evaluation of larger series is urgently needed to characterize early stage substages who can be spared treatment toxicity.

### 4.4. Whether adjuvant chemotherapy improve the prognosis of advanced stage aGCTs?

In advanced stage diseases (stage III and IV) the 5 year DFS and OS were 72% and 80%, respectively,^[64]^ thought about the moderate or high response rate of chemotherapy in early clinical studies, a considerable proportion of the patients received chemotherapy, especially for inoperable tumors or macroscopic residual disease, NCCN guidelines^[21]^ recommend adjuvant chemotherapy for advanced stage disease, but the effect on prognosis remains controversial. The main outcome of studies related to adjuvant chemotherapy in advanced stage aGCTs was summarized in Table 2.

**Table 2:** The main outcome of studies related to adjuvant chemotherapy in advanced stage aGCTs.

**Reference**	**Year**	**Type of research**	**Sample**	**Outcome**
Park et al^[31]^	2012	Retrospective	13	Associated
Ranganath et al^[65]^	2008	Retrospective	34	Not associated
Rzepka et al^[66]^	2012	Retrospective	18	Associated
Al-Badawi et al^[67]^	2002	Retrospective	60	Not associated
Seagle et al^[68]^	2017	Retrospective	2680	Not associated
Meisel et al^[60]^	2015	Retrospective	15	Not associated

Park et al^[31]^ analyzed the role of surgical staging and adjuvant chemotherapy in 13 cases of advanced-stage aGCTs. None of the 6 patients who completed 6 cycles of BEP had recurrence, whereas 5 of the 7 patients (71.4%) who received fewer than 6 cycles of BEP had recurrences and 3 (42.9%) died due to disease. The 5-year DFS rates of these 2 groups were 100% and 50%, respectively (*P* = .022). Optimal debulking followed by 6 cycles of BEP chemotherapy maybe a suitable mode for advanced-stage aGCTs.

Ranganath et al^[65]^ retrospective reviewed the records of 34 patients of aGCTs who were treated over a period of 10 years (1995-2005). Patients who received chemotherapy had a better median DFS than those who did not (60 vs 48 months), but this did not reach statistical significance (*P* = .08).

Rzepka et al^[66]^ retrospectively analyzed patients treated for aGCTs between 1988 and 2008 at the Maria Skłodowska-Curie Memorial Cancer Centre; mean progression free survival was significantly longer in patients treated with the chemotherapy regimen when compared to radiotherapy (148 vs 91 months, respectively; *P* = .028). OS was significantly longer in patients treated with adjuvant chemotherapy vs RTH (165 vs 121 months; *P* = .068).

Al-Badawi et al^[67]^ retrospective reviewed population-based case series identified 60 women with stage IC or greater aGCTs over a 25-year period to assess the use of first-line postoperative chemotherapy in patients with advanced aGCTs. The only statistically significant difference was the presence of macroscopic residual disease (82% vs 22%). There was a trend toward a poorer outcome in the group that received chemotherapy but there was no statistical significant difference. Survival of patients with macroscopic residual disease was not influenced by use of chemotherapy (*P* = .976). It was concluded that the presence of macroscopic residual disease after primary surgery was the most important prognostic factor. Although these patients were more likely to receive postoperative chemotherapy, there was no evidence to document a beneficial effect of systemic therapy in this group.

Seagle et al^[68]^ performed an observational retrospective cohort analysis of 2680 aGCTs of National Cancer Database during 1998 to 2013 and found that receiving adjuvant chemotherapy was not associated with increased survival among women with stages II to IV disease compared to no adjuvant treatment.

Meisel et al^[60]^ performed a retrospective cohort study of aGCTs diagnosed from January 1996 through June 2013 at Memorial Sloan Kettering Cancer Center to assess the role of systemic chemotherapy. Of 118 patients, 10 (8%) received adjuvant chemotherapy (1 of 103 stage I and 9 of 15 stages II-IV patients). Although the numbers were small in this analysis, chemotherapy was not found to improve the recurrence-free interval.

Although the percentage of patients received chemotherapy increased over time, the response rate was desirable, only a few small-sample retrospective studies found that chemotherapy maybe beneficial for DFS or OS for advanced aGCTs. Most of the studies included the largest sample from National Cancer Database and found that chemotherapy was not associated with increased survival and the role of chemotherapy required prospective validation.

### 4.5. Whether adjuvant chemotherapy improve the prognosis of recurrent aGCTs?

Sixteen percent to 23% of aGCTs ultimately developed recurrent disease. Once the tumor recurred, it was fatal in 80% cases.^[69]^ Recurrences were characterized by disseminated peritoneal metastasis. Various treatment options including surgery with/without systemic chemotherapy and/or radiotherapy had been reported for treatment of recurrent aGCTs. There was no standardized management. Cytoreductive surgery for complete resection was cornerstone of treatment. Shim et al^[70]^ retrospectively reviewed recurrent patients and found that the 5-year survival was 60% for patients with residual tumor and 100% for those without residual tumor after secondary surgery. Chua et al^[71]^ demonstrated the feasibility of a repeated peritonectomy to achieve complete cytoreduction in 5 patients with recurrent aGCTs with prolonged DFS, but the role of chemotherapy in recurrent aGCTs was still unknown. A combined modality of treatment, usually involved debulking of the disease followed by radiation or chemotherapy was the norm and might prolong the DFS. The largest study,^[50]^ evaluated BEP as first line therapy in stromal tumors, was by the Gynecologic Oncology Group and found that 69% of patients with advanced disease and 51% with recurrent disease remained progression free over 3 years. The main outcome of studies related to adjuvant chemotherapy in recurrent aGCTs was summarized in Table 3.

**Table 3:** The main outcome of studies related to adjuvant chemotherapy in recurrent aGCTs.

**Reference**	**Year**	**Type of research**	**Sample**	**Outcome**
Gurumurthy et al^[72]^	2014	Systematic Reviews	535	Not sure
Mangili et al^[73]^	2013	Retrospective	35	Not associated
Zhao et al^[74]^	2020	Retrospective	40	Associated
Wang et al^[75]^	2015	Retrospective	44	Not associated
Uygun et al^[76]^	2003	Retrospective	11	Associated

Gurumurthy et al^[72]^ conducted a systematic reviews to evaluate the effectiveness and safety of different chemotherapy used for inoperable disseminated disease or disease with suboptimal cytoreduction, enrolled 5 small retrospective studies, reported no apparent evidence of a difference in OS between surgical approaches, whether a participant underwent lymphadenectomy or received adjuvant chemotherapy or radiotherapy. Although these studies was at high risk of bias and the results should be interpreted with caution.

A retrospective study by Mangili et al^[73]^ enrolled 35 cases of recurrent aGCTs, did not recommend postoperative adjuvant chemotherapy for recurrent aGCTs because 11 of 35 (31.4%) cases recurred despite postoperative adjuvant therapy being performed in 81.8% of all cases. No difference was found in OS among patients receiving or not receiving chemotherapy after secondary surgery at recurrence. However, in most of the previous studies, as much as 70% to 80% of aGCTs recurred and most of them died of disease. By comparison, the recurrence rate in the multicenter Italian trials in ovarian cancer-9 trial appeared to be less than that reported here and in other reports.

Zhao et al^[74]^ retrospective reviewed 40 patients with recurrent aGCTs, found the age at recurrence and postrecurrence therapeutic approach were independent risk factors for death after recurrence. Patients with surgery alone or chemotherapy alone had a significantly higher risk of recurrence and risk of death than those patients with postoperative adjuvant chemotherapy, which suggested that postoperative adjuvant chemotherapy might improve the therapeutic outcomes of recurrent aGCTs.

Wang et al^[75]^ evaluated the long-term outcome of recurrent aGCTs in a large series of patients treated in Taiwanese GOG centers and enrolled 44 patients from 16 medical centers. DFS after the initial operation was the only important predictor for OS, regardless of treatment which suggested that the natural behavior of the tumor was a critical factor for recurrent aGCTs.

Uygun et al^[76]^ evaluated the patients’ characteristics and treatment results for 11 recurrent aGCTs and found an overall response rate of 67% for chemotherapy. There was a marked difference in PFI between patients receiving (24 months) and not receiving (8 months) initial chemotherapy.

According to available small retrospective studies, we are unable to reach valid conclusions to the effectiveness of adjuvant chemotherapy for the management of recurrent aGCTs in clinical practice. Only 2 studies showed improved therapeutic outcomes, in the other studies, no apparent survival advantage was seen, but because all studies were retrospective in nature, at very high risk of bias and likely to be under-powered, given the modest numbers in each study and the multiple adjustments used, the quality of the evidence is low, it is difficult to make meaningful clinical decisions based on these data; thought the poor prognosis for inoperable disease or disease with suboptimal cytoreduction, no other effective alternative treatment, adjuvant chemotherapy maybe an Optable options for those patients, further evidences from good quality prospective studies are needed urgently.

### 4.6. Implications for further research

The available evidences are very limited, further researches are very likely to have an important impact to estimate effect and may alter current practices. Ideally, multinational RCTs are needed urgently. Hormonal therapies^[77]^ are usually tried in advanced stage or recurrent aGCTs. Recurrent chemo-resistant, progressive nonresponding or patients with high surgical risk are ideal candidates for hormone therapy; further evidences from good quality prospective studies are needed; further researches on molecular mechanisms and developing novel targeted medicines may improve patient survival. There were already preclinical studies that found novel target approaches maybe effective for aGCTs, such as targeting mammalian target of rapamycin,^[78]^ vascular endothelial growth factor,^[79]^ phosphatidylinositol-3-kinase serine/threonine kinase pathway,^[80]^ tumor necrosis factor-related apoptosis-inducing ligand,^[81]^ and nuclear factor kB.^[82]^ More studies will be necessary.

## 5. Conclusions

aGCTs are rare ovarian neoplasms with a relatively favorable prognosis characterized by a prolonged natural history and a tendency to late recurrences. Various factors shown to have prognostic significance. Only stage of the disease had been consistently shown in various studies. The percentage of patients receiving chemotherapy are increasing over time, but the benefit of adjuvant chemotherapy is lack of high-grade evidence of prospective study. Based on the current retrospective studies, we still do not have the evidence showing that adjuvant chemotherapy can confer a survival benefit in early stage, advanced stage or recurrent aGCTs with no residual tumor. But for inoperable disseminated disease or disease with suboptimal cytoreduction, adjuvant chemotherapy maybe an Optable options; multinational prospective RCTs are needed to validate the role of adjuvant chemotherapy. Further research on molecular mechanisms and developing novel targeted medicines may improve patient’s survival.

## Acknowledgments

The authors thank the library of The Fifth Affiliated Hospital of Sun Yat-sen University for assistance with literature searching.

## Author contributions

**Data curation:** Yuan Zhuang, Shushan Zhang, Yao Liu.

**Resources:** Shushan Zhang.

**Supervision:** Yuan Zhuang.

**Writing** - **review & editing:** Yuan Zhuang, Hua Yang.

All authors analyzed the literature review. ZY was a major contributor in writing the manuscript, and YH was in charge of the final approval of the version to be published. ZSS and LY searching the literature. All authors read and approved the final manuscript.
